# The animacy effect on free recall is equally large in mixed and pure word lists or pairs

**DOI:** 10.1038/s41598-023-38342-z

**Published:** 2023-07-17

**Authors:** Gesa Fee Komar, Laura Mieth, Axel Buchner, Raoul Bell

**Affiliations:** Department of Experimental Psychology, Heinrich Heine University Düsseldorf, Düsseldorf, Germany

**Keywords:** Psychology, Human behaviour

## Abstract

The cognitive mechanisms underlying the animacy effect on free recall have as yet to be identified. According to the attentional-prioritization account, animate words are better recalled because they recruit more attention at encoding than inanimate words. The account implies that the animacy effect should be larger when animate words are presented together with inanimate words in mixed lists or pairs than when animate and inanimate words are presented separately in pure lists or pairs. The present series of experiments served to systematically test whether list composition or pair composition modulate the animacy effect. In Experiment 1, the animacy effect was compared between mixed and pure lists. In Experiments 2 and 3, the words were presented in mixed or pure pairs to manipulate the direct competition for attention between animate and inanimate words at encoding. While encoding was intentional in Experiments 1 and 2, it was incidental in Experiment 3. In each experiment, a significant animacy effect was obtained, but the effect was equally large in mixed and pure lists or pairs of animate and inanimate words despite considerable sensitivity of the statistical test of the critical interaction. These findings provide evidence against the attentional-prioritization account of the animacy effect.

Other than inanimate objects, animate beings can initiate motion by themselves, grow and reproduce, are capable of mental processes and consist of biological structures that enable biological functions^1^. Based on their evolutionary relevance, it has been postulated that animate beings should have a special status in human cognition^2^. For instance, the animacy effect on free recall (henceforth animacy effect) refers to the finding that words denoting animate beings (henceforth animate words) are better recalled than words denoting inanimate objects (henceforth inanimate words). The animacy effect is robustly found even when animate words have been equated with inanimate words on many other mnemonically relevant word dimensions such as imagery or concreteness (for a review, see^3^). A potential cognitive account of the animacy effect is that animate words recruit more attention at encoding than inanimate words. As will be explicated in more detail below, this *attentional-prioritization account* implies that the difference in recall between animate and inanimate words should be more accentuated when the words are presented in mixed lists composed of both animate and inanimate words than when the words are presented in pure lists composed of either only animate or only inanimate words. As yet, there seems to be only one study^4^ in which the question of whether the animacy effect differs between mixed and pure lists has been addressed. The experiments reported here build on this study and provide a stringent and sensitive test of the attentional-prioritization account of the animacy effect by comparing the animacy effect on free recall between mixed and pure lists (Experiment 1) and between mixed and pure pairs (Experiments 2 and 3) of animate and inanimate words.

The animacy effect on free recall is typically examined by asking participants to learn mixed lists of animate and inanimate words (e.g.,^2,5–7^). Since Nairne et al.’s^2^ discovery, the animacy effect has been robustly replicated in many languages, including English (e.g.,^8,9^), French (e.g.,^10,11^), German (e.g.,^12,13^), Chinese^14^ and Portuguese^5^, using intentional (e.g.,^5,15,16^) and incidental encoding tasks (e.g.,^5,6,17^). The animacy effect was discovered as the result of a functional analysis of memory within the adaptive-memory framework (for a review, see^18^). Memory has been postulated to be tuned to preferably retain animate beings based on the idea that animate beings, as predators, prey or sexual partners, are relevant to the organism’s ultimate goals of survival and reproduction^19^. This functional argument elucidates the potential evolutionary background of the animacy effect, but it does not shed light on the cognitive underpinning of the effect. The functional argument thus should be complemented with an analysis of the cognitive mechanisms underlying the animacy effect (cf.^20^). As yet, progress has mainly been made by ruling out potential cognitive mechanisms. Based on the empirical evidence to date, neither emotional nor mental arousal^12,21^, nor perceived threat (^8^, but see^22^), nor mental imagery^9,23^, nor categorical organization^2,7,9,24^ nor richness of encoding (^13^, but see^15^) provide satisfactory cognitive accounts of the animacy effect.

A potential mechanism that may underlie the animacy effect is that animate words recruit more attention at encoding than inanimate words. A priori, the attentional-prioritization account offers a plausible cognitive mechanism because the allocation of attention at encoding is an important determinant of memory (e.g.,^25^). The idea that the animacy effect is caused by the attentional prioritization of animate relative to inanimate words at encoding has been widely discussed in the literature. For example, Nairne et al.^3^ have proposed, as a potential cognitive account of the animacy effect, “that animate items naturally recruit more attention … which simply maps onto a more accessible memory trace” (p. 26). There is indeed some evidence that animate words recruit more attention than inanimate words: In a Stroop-like task, the processing of the font color of words took longer for animate than for inanimate words, suggesting that animacy recruits attentional resources at the expense of the color-naming task^26^. However, while the findings of Bugaiska et al.^26^ provide evidence for an increased Stroop-like interference by animate words, the study did not include a memory test and thus cannot provide direct evidence on the question of whether the attentional prioritization of animate words is causally related to the animacy effect, that is, the better *memory* for animate than for inanimate words. To demonstrate such a causal relationship, it is necessary to show that manipulations that are expected to enable or disable the attentional prioritization of animate words affect the animacy effect. To illustrate the importance of experimental manipulations of variables affecting the construct in question, consider, for instance, the richness-of-encoding account of the animacy effect. Initial correlational findings have shown that animate words are associated with a richer idea formation than inanimate words in idea-generation tasks^6,11^ and that animate words are more richly represented in memory^10,15,27–29^. However, correlation does not imply causation. Therefore, experimental manipulations of richness of encoding were necessary to test whether different levels of richness of encoding would affect the animacy effect. The fact that the animacy effect remained unaffected by experimental manipulations of richness of encoding has considerably weakened richness of encoding as the primary cognitive mechanism underlying the animacy effect^13^. Analogously, the finding that animate words interfere more with color naming than inanimate words in a Stroop-like task^26^ is an intriguing phenomenon in itself, but experimental manipulations of attention are necessary to test the causal contribution of attentional prioritization to the animacy effect.

Studies in which a dual-task paradigm (see^25^) was used to test whether the animacy effect is modulated by attentional load provided mixed evidence regarding the role of attention in the animacy effect: In two studies, the animacy effect remained unaffected by whether or not attention at encoding was divided between the encoding task and a secondary task^15,30^. By contrast, Leding^8^ observed that the animacy effect was significantly decreased—but not completely eliminated—when a secondary task had to be performed compared to when no secondary task had to be performed. Furthermore, in one of the three dual-task experiments of Bonin et al.^30^, performance in the secondary task decreased when the task had to be performed while animate words were presented, indicating that animate words recruited more attentional resources at the expense of the secondary task than inanimate words. The mixed evidence available so far thus does not allow for a firm rejection or confirmation of the attentional-prioritization account. Therefore, further empirical tests of the account are necessary.

Another way to test the attentional-prioritization account is to examine whether the animacy effect differs between mixed lists composed of both animate and inanimate words and pure lists composed of either only animate or only inanimate words. Just as in the Stroop-like task and the dual-task paradigm, this test rests on the assumption that attention is a limited resource. The attentional-prioritization account implies that there is an asymmetry in the allocation of these limited attentional resources between animate and inanimate words such that the animate words are prioritized at the expense of the inanimate words. Many other mnemonic effects have been demonstrated to differ as a function of whether mixed or pure lists are used^31^. For instance, robust list-composition effects have been obtained when examining the effects of emotional arousal on the recognition and the free recall of words and pictures. Emotionally arousing stimuli are better remembered than neutral stimuli when emotionally arousing stimuli are presented together with neutral stimuli in mixed lists, but the effect of emotional arousal on memory is often severely reduced or even completely eliminated when emotionally arousing and neutral stimuli are presented separately in pure lists^32–34^. A possible explanation of these list-composition effects is that attentional resources are allocated asymmetrically between emotionally arousing and neutral stimuli in mixed lists^35,36^: The emotionally arousing stimuli are prioritized at the expense of the neutral stimuli in the same list so that the emotionally arousing stimuli are better remembered at the expense of the neutral stimuli. This attentional prioritization results in an accentuation of the effect of emotional arousal on memory when using mixed versus pure lists.

Parallel to the reasoning above, the attentional-prioritization account of the animacy effect implies that the animacy effect should be larger in mixed lists than in pure lists. The use of a pure-list design has been offered as a reason for the absence of an animacy effect on visual statistical learning^37^, but due to the predominance of the mixed-list design when examining the animacy effect on free recall, there is only sparse evidence on whether the animacy effect differs between mixed and pure lists. Initial evidence was provided by Popp and Serra^4^. Their experiment included, among other conditions, a comparison of the animacy effect on free recall between mixed and pure lists. Their results were, however, somewhat ambiguous. At the level of statistical inference, there was no significant interaction between animacy and list composition, but at the descriptive level, the animacy effect was about twice as large in the mixed lists compared to the pure lists. This data pattern raises the question of whether the sample size of *N* = 64 participants might have been too small to detect the critical interaction between animacy and list composition. Moreover, the complexity of the experimental design might have induced interference because participants were required to perform free-recall and cued-recall tests in a repeated-measures design.

The present Experiment 1 was designed as a replication of Popp and Serra’s^4^ comparison of the animacy effect between mixed and pure lists with increased sensitivity in a less complex experimental design that focused only on the critical interaction between animacy and list composition on free recall. In the present Experiments 2 and 3, the words were presented simultaneously in mixed pairs composed of one animate and one inanimate word or in pure pairs composed of either two animate or two inanimate words to manipulate the competition for attention at the time of presentation. Whereas intentional encoding tasks were used in Experiments 1 and 2, Experiment 3 served to test whether animate words may receive a stronger attentional priority relative to inanimate words in an incidental encoding task. The attentional-prioritization account of the animacy effect implies that the animacy effect should be larger when animate words are presented together with inanimate words in mixed lists or pairs than when animate and inanimate words are presented separately in pure lists or pairs.

## Experiment 1

### Method

#### Participants

The experiment was conducted online using SoSci Survey^38^. Participants were allowed to use a desktop or laptop computer, not a tablet or smartphone. To make use of the original English word material made openly available by Popp and Serra^4^, participants were recruited in the United Kingdom from the research panels of the ISO-20252:2019-certified online-access-panel provider respondi AG (https://www.respondi.com).

We aimed to collect 500 complete data sets (and up to 50 additional complete data sets to compensate for exclusions) and stopped data collection once this criterion was reached. Of 637 participants who had started the intentional encoding task, 94 participants had to be excluded because they either did not complete the experiment or withdrew their consent to the use of their data. The data of 27 participants were excluded because these participants did not recall any of the presented words. Eighteen participants indicated issues with understanding English, reading the text on the screen, complying with instructions or with the display of the stimuli. Following a recommendation of Elliott et al.^39^, the data of these participants were included in the final analysis; their exclusion would not have changed any statistical conclusions. The final sample of Experiment 1, characterized by diversified levels of education and good English language proficiency, consisted of 516 participants (241 female, 274 male, 1 nonbinary) aged between 18 and 85 (*M* = 45, *SD* = 16) years. The participants were randomly assigned to either the mixed-lists group (*n* = 260) or the pure-lists group (*n* = 256). A sensitivity analysis with G*Power^40^ showed that, with a sample size of *N* = 516 and α = 0.05, an interaction between animacy and list composition (that is, a variation of the animacy effect as a function of whether free recall was measured in the mixed-lists group or in the pure-lists group) on free recall as small as $\upeta_{\text p}^2$ = 0.02 could be detected with a statistical power of 1 − β = 0.95. Participants received a small monetary compensation for participating.

#### Ethics statement

In all experiments reported here, participants gave written informed consent prior to participation. The experiments were conducted in line with the Declaration of Helsinki and belonged to a series of experiments for which approval was obtained from the ethics committee of the Faculty of Mathematics and Natural Sciences at Heinrich Heine University Düsseldorf.

#### Materials, design and procedure

We used the English word material of Popp and Serra^4^. The pool consists of 84 animate words and 84 inanimate words that were matched on imagery, concreteness, word frequency and the number of letters (for details, see^4^).

The experiment had a mixed design with the within-subjects factor animacy (animate, inanimate) and the between-subjects factor list composition (mixed, pure). For each participant, two lists were created by randomly selecting words (without replacement) from the word pool. For participants in the pure-lists group, one list was composed of 16 randomly-ordered animate words and the other list was composed of 16 randomly-ordered inanimate words. The order of list presentation was counterbalanced across participants. For participants in the mixed-lists group, each of the two lists was composed of 8 animate and 8 inanimate words in a random order.

The procedure was similar to that of Experiment 1 of Popp and Serra^4^ with the exception that free recall served as the only memory test. In the intentional encoding task, participants were informed that their task was to study two lists of nouns. Participants knew that every noun would be presented for 5 s and that it would not be possible to pause or to repeat the presentation. Thus, words were presented one after another as is typical for experiments in which the effect of animacy on free recall is examined (e.g.,^2,5–7,12,13,16^). Participants were informed that after each list, they would be asked to recall the nouns of that list in any order. Words of each list were shown in black bold 36-point Arial font at the center of the browser window. After all words of the first list had been presented, participants were instructed to recall as many of the presented nouns as possible. They were reminded that the order of the nouns was not important. Participants were asked to type each noun into a separate text field. There were 16 text fields, matching the number of words presented at encoding. When participants were sure that they could not recall any more of the nouns, they clicked a “finish memory test” button which was possible regardless of the number of words that had been recalled. The same procedure was then repeated for the second list, starting with the presentation of the list and followed by the free recall of the nouns. Before the presentation of the second list and before the second memory test, it was emphasized that the nouns of the first list were not supposed to be recalled in the second memory test.

At the end of the experiment, participants were thanked for their participation. After being instructed to provide honest answers so that reliable conclusions could be drawn from the results and being told that their answers would not have any consequences for them (cf.^41^), participants were asked whether they had complied with the instructions and whether all information had been displayed correctly. Directly after these control questions, participants were asked whether we would be allowed to use their data in an anonymized form for the data analysis, thereby giving participants the opportunity to revoke their consent, given prior to participation, by clicking on a “No, I withdraw the consent to the use of my data” option. The median duration of the experiment was 7 min.

### Results

Free recall was measured by calculating the proportion of list words that were correctly recalled. A word was scored as correctly recalled only if it belonged to the immediately preceding word list. The semi-automated scoring of the free-recall data followed a two-step procedure. First, a computer program scored the exact matches between the recalled words and the list words. The remaining words were manually evaluated by three human raters. Obvious spelling mistakes or the use of the plural forms of the words were scored as correct. The manually evaluated words comprised about 5 % of the correctly recalled words.

**Figure 1:**
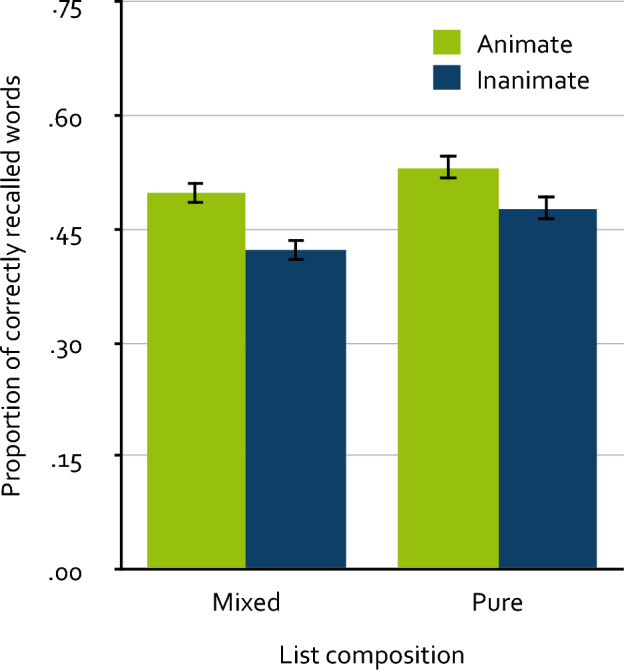
Mean proportion of correctly recalled words as a function of animacy and list composition in Experiment 1. Participants intentionally encoded either two mixed lists composed of both animate and inanimate words or one pure list composed of only animate words and one pure list composed of only inanimate words. The proportion of correctly recalled words refers to words recalled within the two memory tests combined. The error bars represent the standard errors of the means.

Figure 1 displays the mean proportion of correctly recalled words as a function of animacy and list composition. The α level was set to 0.05 for all statistical tests. A 2 × 2 mixed analysis of variance (ANOVA) showed a significant recall advantage of animate over inanimate words, *F*(1, 514) = 68.27, *p* < 0.001, $\upeta_{\text p}^2$ = 0.12. Words presented in pure lists were recalled significantly better than words presented in mixed lists, *F*(1, 514) = 4.79, *p* = 0.029, $\upeta_{\text p}^2$ = 0.01. The critical interaction between animacy and list composition was not significant, *F*(1, 514) = 1.65, *p* = 0.199, $\upeta_{\text p}^2$ < 0.01, supporting the conclusion that the animacy effect does not differ between mixed and pure lists.

The mean number of intrusions is reported in Table 1. Intrusions from the first to the second list were distinguished from extra-experimental intrusions. The extra-experimental intrusions were categorized as animate, inanimate or uncategorizable by the three raters; inconsistencies among the raters were resolved by discussion. Intrusions occurred extremely rarely which precludes the use of inferential statistics for all experiments reported here.

**Table 1:** Mean number of intrusions as a function of animacy in Experiments 1, 2 and 3.

	Intrusion type		Category					
			Animate		Inanimate		Uncategorizable	
Experiment 1	First-to-second list	Mixed lists	0.08	(0.02)	0.05	(0.02)	–	–
		Pure lists	< 0.01	(< 0.01)	0.00	(0.00)	–	–
	Extra-experimental	Mixed lists	0.14	(0.03)	0.25	(0.04)	0.12	(0.07)
		Pure lists	0.21	(0.04)	0.27	(0.05)	0.23	(0.11)
Experiment 2	Extra-experimental		0.22	(0.02)	0.26	(0.03)	0.05	(0.01)
Experiment 3	Extra-experimental		0.10	(0.02)	0.24	(0.03)	0.08	(0.02)

In Experiment 1, we distinguished between intrusions from the first to the second list and extra-experimental intrusions, calculated separately for the levels of the list-composition factor. In Experiments 1, 2 and 3, extra-experimental intrusions refer to falsely reported words that were not presented at encoding. The values in parentheses represent the standard errors of the means.

### Discussion

Despite a large sample size of *N* = 516 that ensured a high sensitivity to detect an interaction between animacy and list composition given α = β = 0.05, the animacy effect was found to be equally large in mixed and pure lists; the sample effect size associated with the critical interaction was $\upeta_{\text p}^2$ < 0.01. The results thus allow us to confirm, with a higher sensitivity of the statistical test, the conclusion of Popp and Serra^4^ that there is no interaction between animacy and list composition on free recall. The data pattern provides evidence against the attentional-prioritization account according to which the animacy effect is caused by animate words recruiting attention at the expense of inanimate words.

## Experiment 2

In Experiment 1, all words were presented sequentially. This implies that the animate and inanimate words in mixed lists did not directly compete for resource-constraint processes at the time they were presented but only for resource-constraint processes that extended beyond the immediate presentation of the words. This raises the question of whether animate words would recruit attentional resources at the expense of the encoding of inanimate words when animate and inanimate words were presented simultaneously so that they could compete for resource-constraint processes at the time of presentation (cf.^42–44^). To test this hypothesis in Experiment 2, the words were presented simultaneously in mixed pairs composed of one animate and one inanimate word or in pure pairs composed of either two animate or two inanimate words. If the encoding of the animate words benefits from attentional prioritization in mixed pairs, the free recall of animate words should improve at the expense of the free recall of inanimate words. In pure pairs, competition for resource-constraint processes at the time of presentation could occur only between words of the same animacy status with the result that the encoding of the animate words could not be prioritized at the expense of the encoding of the inanimate words. The critical test was whether there is an interaction between animacy and pair composition on free recall. According to the attentional-prioritization account, the animacy effect should be larger when animate words are presented together with inanimate words in mixed pairs than when animate and inanimate words are presented separately in pure pairs.

### Method

#### Participants

Participants who had not participated in Experiment 1 were recruited in the same way as in Experiment 1. Of 645 participants who had started the intentional encoding task, 101 participants had to be excluded because they either did not complete the experiment or withdrew their consent to the use of their data. The data of 36 participants were excluded because these participants did not recall any of the presented words. Eleven participants indicated issues with understanding English, reading the text on the screen, complying with instructions or with the display of the stimuli. Following a recommendation of Elliott et al.^39^, the data of these participants were included in the final analysis; their exclusion would not have changed any statistical conclusions. The final sample consisted of 508 participants (318 female, 187 male, 3 nonbinary) aged between 18 and 72 (*M* = 49, *SD* = 13) years. A sensitivity analysis with G*Power^40^ showed that, with a sample size of *N* = 508 and α = 0.05, an interaction between animacy and pair composition (that is, a variation of the animacy effect as a function of whether the word pairs were mixed or pure) on free recall as small as $\upeta_{\text p}^2$ = 0.03 could be detected with a statistical power of 1 − β = 0.95.

#### Materials, design and procedure

As in Experiment 1, a random selection of 16 animate and 16 inanimate words was drawn from the word pool of Popp and Serra^4^ for each participant. Other than in Experiment 1, the words were presented simultaneously in mixed pairs composed of one animate and one inanimate word or in pure pairs composed of either two animate or two inanimate words. The experiment had a within-subjects design with the factors animacy (animate, inanimate) and pair composition (mixed, pure). For each participant, the 16 animate and 16 inanimate words were randomly assigned to 8 mixed pairs and 8 pure pairs (4 pairs of animate words and 4 pairs of inanimate words) that were presented in a random order. The position (left or right) of animate and inanimate words in mixed pairs was counterbalanced.

In the intentional encoding task, participants were instructed to study several nouns. Participants knew that two nouns would be presented together for 5 s each and that it would not be possible to pause or to repeat the presentation. Participants were informed that, later on, they would be asked to recall all individual nouns in any order. Word pairs were shown in black bold 36-point Arial font at the center of the browser window. After all words had been presented, participants were instructed to recall as many of the presented nouns as possible. They were reminded that the order of the nouns was not important and that it would not be necessary to group nouns which had been presented together. There were 32 text fields, matching the number of words presented at encoding. Participants were asked to type each noun into a separate text field. When they were sure that they could not recall any more of the nouns, they clicked a “finish memory test” button. This was possible regardless of the number of words that had been recalled. The median duration of the experiment was 4 min.

### Results

**Figure 2:**
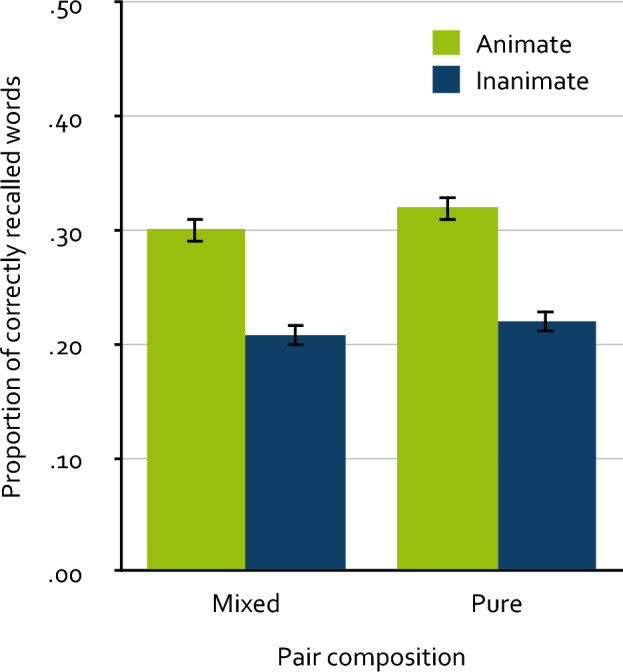
Mean proportion of correctly recalled words as a function of animacy and pair composition in Experiment 2. Participants intentionally encoded words presented in pairs of the configurations animate–inanimate (mixed), inanimate–animate (mixed), animate–animate (pure) and inanimate–inanimate (pure). The proportion of correctly recalled words refers to single words recalled within a single memory test. The error bars represent the standard errors of the means.

The free-recall data were scored with the same semi-automated procedure as in Experiment 1. The manually evaluated words comprised about 6 % of the correctly recalled words. Figure 2 displays the mean proportion of correctly recalled words as a function of animacy and pair composition. A 2 × 2 repeated-measures ANOVA showed that animate words were recalled significantly better than inanimate words, *F*(1, 507) = 233.78, *p* < 0.001, $\upeta_{\text p}^2$ = 0.32. Furthermore, words presented in pure pairs were recalled significantly better than words presented in mixed pairs, *F*(1, 507) = 4.30, *p* = 0.039, $\upeta_{\text p}^2$ = 0.01. However, the critical interaction between animacy and pair composition was not significant, *F*(1, 507) = 0.28, *p* = 0.598, $\upeta_{\text p}^2$ < 0.01, leading to the conclusion that the animacy effect does not differ between mixed and pure pairs.

The mean number of extra-experimental intrusions is reported in Table 1. Intrusions were again extremely rare.

### Discussion

Despite the large sample size of *N* = 508 that ensured a high sensitivity to detect an interaction between animacy and pair composition given α = β = 0.05, the animacy effect was equally large in mixed and pure pairs; the sample effect size associated with the critical interaction was $\upeta_{\text p}^2$ < 0.01. This held true even though the animate and inanimate words were shown simultaneously in mixed pairs which should have caused competition for attention at the time of presentation. The data pattern thus provides evidence against the attentional-prioritization account according to which the animacy effect is caused by animate words recruiting attention at the expense of inanimate words at encoding.

## Experiment 3

Parallel to Popp and Serra^4^, intentional encoding tasks were used in Experiments 1 and 2. However, it seems possible that an asymmetry in the allocation of attentional resources between animate and inanimate words may play a greater role in incidental encoding tasks than in intentional encoding tasks. In intentional encoding tasks, participants are required to attend to all words that have to be recalled later, regardless of their animacy status. In incidental encoding tasks in which the participants do not know that all words have to be recalled later, intrinsic properties of the words such as the animacy status of the words may be more likely to have an impact on the allocation of attentional resources at encoding. Consistent with this hypothesis, Félix et al.^5^ reported that the animacy effect was larger after incidental encoding than after intentional encoding. Félix et al.^5^ discussed, as a potential explanation of this pattern, that animate words may receive a stronger attentional priority relative to inanimate words in an incidental encoding task than in an intentional encoding task. To test this possibility, an incidental encoding task was used in Experiment 3. The predictions derived from the attentional-prioritization account of the animacy effect were the same as those in Experiment 2: The critical test was whether there is an interaction between animacy and pair composition on free recall. If the animacy effect is caused by animate words recruiting attention at the expense of inanimate words at the time of presentation, the animacy effect should be larger in mixed pairs composed of one animate and one inanimate word than in pure pairs composed of either two animate or two inanimate words.

### Method

#### Participants

Participants who had not participated in either Experiment 1 or Experiment 2 were recruited as before. Of 635 participants who had started the incidental encoding task, 88 participants had to be excluded because they either did not complete the experiment or withdrew their consent to the use of their data. The data of 109 participants were excluded because these participants did not recall any of the presented words. The data of one participant’s repeated participation were excluded as well. Fourteen participants indicated issues with understanding English, reading the text on the screen, complying with instructions or with the display of the stimuli. Following a recommendation of Elliott et al.^39^, the data of these participants were included in the final analysis; their exclusion would not have changed any statistical conclusions. The final sample consisted of 437 participants (157 female, 277 male, 3 nonbinary) aged between 18 and 85 (*M* = 44, *SD* = 16) years. A sensitivity analysis with G*Power^40^ showed that, with a sample size of *N* = 437 and α = 0.05, an interaction between animacy and pair composition (that is, a variation of the animacy effect as a function of whether the word pairs were mixed or pure) on free recall as small as $\upeta_{\text p}^2$ = 0.03 could be detected with a statistical power of 1 − β = 0.95.

#### Materials, design and procedure

For each participant, animate and inanimate words were selected from the word pool of Popp and Serra^4^ and assigned to word pairs as in Experiment 2. Parallel to Experiment 2, the experiment had a within-subjects design with the factors animacy (animate, inanimate) and pair composition (mixed, pure). Other than in Experiment 2, an incidental encoding task was used. Participants were asked to count the number of letters of both nouns together and to type the total number of letters into a single text field. Participants were asked to complete this task as accurately as possible. The pools of animate and inanimate words from which a random selection of 16 animate and 16 inanimate words was drawn for each participant were equated in the number of letters^4^. The proportion of correct responses in the letter-counting task did not significantly differ among the mixed pairs of one animate and one inanimate word (*M* = 0.93, *SE* = 0.01), the pure pairs of two animate words (*M* = 0.94, *SE* = 0.01) and the pure pairs of two inanimate words (*M* = 0.94, *SE* = 0.01), *F*(2, 435) = 1.98, *p* = 0.140, $\upeta_{\text p}^2$ = 0.01. Recording of the response times began when the word pair was presented and ended when the participant clicked on a “next” button to proceed. The mean response time did not significantly differ among the mixed pairs of one animate and one inanimate word (*M* = 13.58 s, *SE* = 1.72), the pure pairs of two animate words (*M* = 10.95 s, *SE* = 0.42) and the pure pairs of two inanimate words (*M* = 12.00 s, *SE* = 0.86), *F*(2, 435) = 1.82, *p* = 0.164, $\upeta_{\text p}^2$ = 0.01. With the exception of being unannounced, the free-recall test was identical to that used in Experiment 2. The median duration of the experiment was 5 min.

### Results

**Figure 3:**
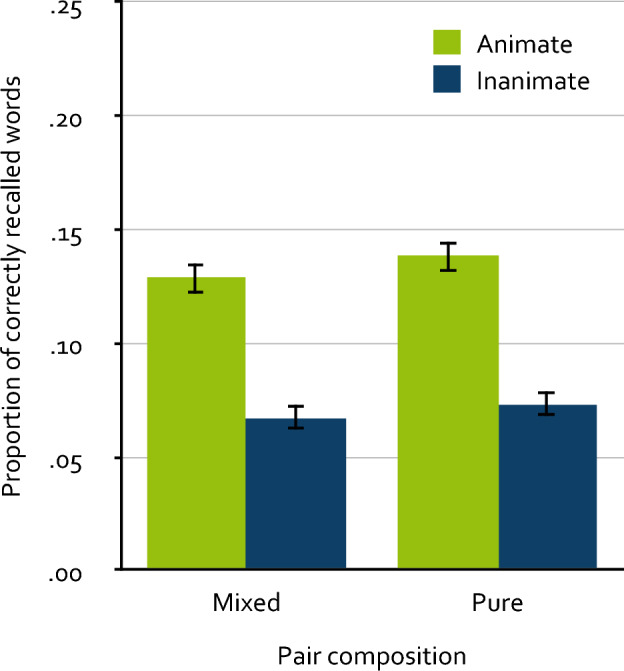
Mean proportion of correctly recalled words as a function of animacy and pair composition in Experiment 3. Participants incidentally encoded words presented in pairs of the configurations animate–inanimate (mixed), inanimate–animate (mixed), animate–animate (pure) and inanimate–inanimate (pure). The proportion of correctly recalled words refers to single words recalled within a single memory test. The error bars represent the standard errors of the means.

The free-recall data were scored with the same semi-automated procedure as in Experiments 1 and 2. The manually evaluated words comprised about 4 % of the correctly recalled words. Figure 3 displays the mean proportion of correctly recalled words as a function of animacy and pair composition. Due to the incidental encoding task, participants recalled fewer words overall than in Experiment 2 at the descriptive level. A 2 × 2 repeated-measures ANOVA showed that animate words were recalled significantly better than inanimate words, *F*(1, 436) = 183.03, *p* < 0.001, $\upeta_{\text p}^2$ = 0.30. Free recall did not significantly differ between words presented in mixed pairs and words presented in pure pairs, *F*(1, 436) = 1.80, *p* = 0.181, $\upeta_{\text p}^2$ < 0.01. Importantly, the critical interaction between animacy and pair composition was not significant, *F*(1, 436) = 0.12, *p* = 0.730, $\upeta_{\text p}^2$ < 0.01.

The mean number of extra-experimental intrusions is reported in Table 1. Intrusions were again extremely rare.

### Discussion

Despite the large sample size of *N* = 437 that ensured a high sensitivity to detect an interaction between animacy and pair composition given α = β = 0.05, the animacy effect was equally large in mixed and pure pairs; the sample effect size associated with the critical interaction was $\upeta_{\text p}^2$ < 0.01. This held true even though encoding was incidental in Experiment 3. It thus can be concluded that the animacy effect is not affected by the presence or absence of direct competition for attention between animate and inanimate words at encoding, irrespective of whether encoding is intentional or incidental. This rules out the possibility that an asymmetry in the allocation of attentional resources between animate and inanimate words in mixed pairs was overshadowed by the participants’ intentional memorization strategies and instead further strengthens the general conclusion that the animacy effect is not caused by the attentional prioritization of animate words at encoding.

## General discussion

While the adaptive-memory framework^18,19^ provides a potential account of the evolutionary background of the animacy effect on free recall, the cognitive mechanisms underlying the animacy effect have as yet to be identified. The present series of experiments served to test the attentional-prioritization account according to which there is an asymmetry in the allocation of attentional resources between animate and inanimate words such that animate words are prioritized at the expense of inanimate words. A straightforward implication of this account is that the animacy effect should be larger in mixed lists in which animate words compete with inanimate words for processing resources than in pure lists in which there is no direct competition between animate and inanimate words. Given that almost all previous studies have used mixed lists (e.g.,^2,5–7,10,12,13^), it was unclear whether the animacy effect would indeed turn out to be smaller in pure lists. The starting point of the present investigation was the observation that in one previous study in which the animacy effect was compared between mixed and pure lists^4^, the results appeared to be ambiguous: At the descriptive level, the animacy effect was about twice as large in the mixed lists compared to the pure lists, but the critical interaction between animacy and list composition was not statistically significant. This observation raised the question of whether the interaction between animacy and list composition would turn out to be statistically significant when the sensitivity of the statistical test was enhanced by increasing the sample size. Therefore, we conducted an experiment in which the sample size was increased from *N* = 64 in the original experiment of Popp and Serra^4^ to *N* = 516 in the present Experiment 1. The results of Experiment 1 confirm that the animacy effect is equally large in mixed and pure word lists. The substantial increase in sample size relative to the original experiment of Popp and Serra^4^ greatly reduces the risk that the interaction between animacy and list composition was not detected due to a lack of sensitivity of the statistical test of this interaction. The results of Experiment 1 thus weaken the attentional-prioritization account of the animacy effect.

A possible limitation of the experimental test adopted in Experiment 1 was that the words were presented sequentially which implies that, in mixed lists, animate and inanimate words could compete only for resource-constraint processes that extended beyond their immediate presentation. In Experiments 2 and 3, the words were presented in mixed pairs composed of one animate and one inanimate word or in pure pairs composed of either two animate or two inanimate words. Simultaneously presenting animate and inanimate words in mixed pairs should have accentuated the direct competition for attention between animate and inanimate words at encoding compared to the sequential presentation that was used in Experiment 1 (cf.^42–44^). Nevertheless, no interaction between animacy and pair composition was found. In Experiments 1 and 2, intentional encoding tasks were used. It could have been argued that requiring participants to memorize and thus to attend to all words regardless of their animacy status may have reduced the effect of animacy on attentional prioritization (cf.^5^). Therefore, Experiment 3 was designed to test whether animate words recruit attention at the expense of inanimate words when an incidental encoding task is used. Indeed, intentional and incidental encoding led to somewhat different outcomes with regard to the effects of list and pair composition on free recall. In Experiments 1 and 2 in which intentional encoding tasks were used, list composition and pair composition, respectively, had a main effect on free recall, suggesting that pure lists and pairs lend themselves somewhat better to intentional memorization strategies than mixed lists and pairs. The main effect of pair composition was absent in Experiment 3. However, despite using a letter-counting task, the animacy effect was equally large in mixed and pure pairs. The sample effect size $\upeta_{\text p}^2$ associated with the critical interaction between animacy and list composition or pair composition was smaller than 0.01 in each of the three experiments which further supports the conclusion that the present findings provide consistent evidence against the attentional-prioritization account.

The present series of experiments extends findings that have already weakened the attentional-prioritization account of the animacy effect. In three studies in which a dual-task paradigm was used, the animacy effect did not depend on attentional load^15,30^ or was only decreased but not completely eliminated when a secondary task had to be performed compared to when no secondary task had to be performed^8^. These findings were interpreted as providing evidence against a strong role of attentional prioritization in the animacy effect. For instance, Bonin et al.^30^ concluded that their results provide “no evidence suggesting that attentional resources are allocated differently to animates compared to inanimates” (p. 380). The strongest evidence in favor of the attentional-prioritization account has been offered by the study of Bugaiska et al.^26^ who found that naming the font color of animate words took longer than naming the font color of inanimate words in a Stroop-like task. However, the study did not include a memory test and thus could not provide direct evidence on the relationship between impaired color naming of animate words and the robustly found recall advantage of animate over inanimate words. To advance our understanding of the cognitive mechanisms underlying the animacy effect, the necessity of experimental manipulations targeting the causal factor of interest has previously been emphasized^13^. This point is readily illustrated using the richness-of-encoding account. It has been counted as initial support for this account that animate words, compared to inanimate words, stimulate a richer idea formation in idea-generation tasks^6,11^ and have richer representations in memory^10,15,27–29^. However, the fact that experimental manipulations targeting richness of encoding did not modulate the recall advantage of animate over inanimate words challenged the theory that richness of encoding is the primary cognitive mechanism underlying the animacy effect^13^. In a similar vein, the results of the present experimental manipulations challenge the theory that attentional prioritization is the primary cognitive mechanism underlying the animacy effect.

In the present series of experiments, we relied on the original English word material of Popp and Serra^4^ because Experiment 1 was designed as a replication of Popp and Serra’s^4^ results obtained in free recall with increased sensitivity of the statistical test. Systematically varying one particular aspect of the procedure such as the sample size while holding other aspects such as the word material constant has the advantage of providing a contribution to the cumulative understanding of the subject matter by facilitating the interpretation of the results. It remains up to future studies to test whether the present finding that the animacy effect is equally large in mixed and pure word lists or pairs generalizes across different word materials (cf.^16,45^).

Encoding conditions under which animacy effects have been observed are diverse and range from intentional encoding tasks (e.g.,^2,5,7,8,15,16,30^) to various incidental encoding tasks that stimulate shallow levels of processing^13,46^ or deep levels of processing, including tasks demanding animacy categorization (e.g.,^10,23,30^), pleasantness rating^5,9,17,46^, idea generation^6,11,13^ and survival processing^13,17,46^. The present series of experiments further underlines the robustness of the animacy effect in showing that the animacy effect is not limited to mixed lists but generalizes to pure lists and is equally large in mixed pairs composed of one animate and one inanimate word and pure pairs composed of either two animate or two inanimate words presented together at encoding. At the theoretical level, progress in understanding the animacy effect has as yet primarily been made by ruling out potential cognitive accounts of the effect. For example, theories attributing the animacy effect to emotional or mental arousal^12,21^, perceived threat (^8^, but see^22^), mental imagery^9,23^, categorical organization^24^ or richness of encoding^13^ have been disconfirmed by the available empirical evidence. The present series of experiments lines up well with these previous studies in that its main contribution is to provide evidence against the widely discussed attentional-prioritization account of the animacy effect. It is thus up to future studies to identify the cognitive mechanism or combinations of cognitive mechanisms that underlie the animacy effect.

## Data Availability

The data of all experiments reported here and supplementary Bayesian analyses of the results are available at the project page of the Open Science Framework, https://osf.io/x4am5/.
